# The causal relationship between cathepsins and digestive system tumors: a Mendelian randomization study

**DOI:** 10.3389/fonc.2024.1365138

**Published:** 2024-03-25

**Authors:** Xupeng Huang, Houbo Deng, Bo Zhang, Kuisong Wang, Yi Qu, Ting Li, Tiejun Liu

**Affiliations:** ^1^ Graduate School, Changchun University of Traditional Chinese Medicine, Changchun, China; ^2^ Department of Hepatology, First Affiliated Hospital to Changchun University of Chinese Medicine, Changchun, China

**Keywords:** Mendelian randomization, cathepsins, digestive system tumors, genome-wide association, causal relationship

## Abstract

**Background:**

Multiple studies have confirmed the significant role of cathepsins in the development and progression of digestive system tumors. However, further investigation is needed to determine the causal relationships.

**Methods:**

We conducted a two-sample bidirectional Mendelian randomization (MR) study using pooled data from a genome-wide association study (GWAS) to assess the causal associations between nine cathepsins (cathepsin B, E, F, G, H, L2, O, S, and Z) and six types of digestive system tumors, including hepatocellular carcinoma (HCC), pancreatic cancer (PCa), biliary tract cancer (BTC), colorectal cancer (CRC), gastric carcinoma (GC), and esophageal cancer (EC). We employed the following methods including inverse variance weighting (IVW), MR-Egger, weighted median (WM), Cochran’s Q, MR-PRESSO, MR-Egger intercept test and leave-one-out sensitivity analysis. The STROBE-MR checklist for the reporting of MR studies was used in this study.

**Results:**

The risk of HCC increased with high levels of cathepsin G (IVW: p = 0.029, odds ratio (OR) = 1.369, 95% confidence interval (CI) = 1.033-1.814). Similarly, BTC was associated with elevated cathepsin B levels (IVW: p = 0.025, OR = 1.693, 95% CI = 1.070-2.681). Conversely, a reduction in PCa risk was associated with increased cathepsin H levels (IVW: p = 0.027, OR = 0.896, 95% CI = 0.812-0.988). Lastly, high levels of cathepsin L2 were found to lower the risk of CRC (IVW: p = 0.034, OR = 0.814, 95% CI = 0.674-0.985).

**Conclusion:**

Our findings confirm the causal relationship between cathepsins and digestive system tumors, which can offer valuable insights for the diagnosis and treatment of digestive system tumors.

## 1 Introduction

Digestive system tumors are a significant contributor to cancer-related deaths globally. Their incidence and mortality rates have been increasing consistently, presenting a significant challenge to human health (1). The primary types of digestive system tumors include hepatocellular carcinoma (HCC), pancreatic cancer (PCa), biliary tract cancer (BTC), colorectal cancer (CRC), gastric carcinoma (GC), and esophageal cancer (EC) (2). The occurrence and development of digestive system tumors are complex processes that involve multiple risk factors. One important factor in the formation of these tumors is the ability of carcinoma cells to sustain internal homeostasis (3). Proteins have a critical function in maintaining the metabolic equilibrium of cancer cells, with the activity of the proteolytic system being especially important for the proliferation of cancer cells (4). Cathepsins are lysosomal proteolytic enzymes that are responsible for maintaining cellular homeostasis. They primarily function as endopeptidases within the lysosomal vesicles of normal cells (5). Cathepsins are involved in various physiological processes such as protein turnover, differentiation, and apoptosis. They also play important roles in signaling cellular stress, breaking down the extracellular matrix, causing lysosome-mediated cell death, and have been associated with the progression of a diversity of diseases, including malignancies (6).

According to previous studies, cathepsins may contribute to the development and progression of diverse types of cancers, including breast cancer (7, 8), bladder cancer (9), thyroid cancer (10), and others. Notably, multiple cancer types have been linked to cathepsin B (11). A recent Mendelian randomization (MR) study revealed that elevated levels of cathepsin H may contribute to an increased risk of lung cancer (12). Previous observational studies have indicated a correlation between cathepsins and tumors in the digestive system, specifically cathepsin L (13) and cathepsin W (14), which have been linked to a poor prognosis for PCa. Cathepsin B has been linked to HCC (15), PCa (16, 17), BTC (18, 19), and CRC (20, 21). Furthermore, Cathepsin L (22) plays a significant role in angiogenesis of GC. Due to the lack of clear causal relationship between cathepsins and digestive system tumors in previous observational studies, we employed MR analysis. MR is a statistical method commonly used in genetic epidemiology studies, which combines data from genome-wide association studies (GWAS). One of the key advantages of MR is its ability to handle confounding factors and eradicate reverse causality by using genetic variants as instrumental variables (IVs) (23).

The objective of this research was to examine the correlation between cathepsins and digestive system tumors by analyzing genetic variants associated with nine cathepsins and six digestive system tumors obtained from a large GWAS. The analysis was conducted using a two-sample bidirectional MR approach. Our research findings significantly augment the comprehension of the causal relationship between cathepsins and these digestive system tumors.

## 2 Materials and methods

### 2.1 Data sources

The MR analysis of nine cathepsin levels in this investigation obtained the genetic tools from the INTERVAL study, which comprised 3301 Europeans (24). Every contributor was obligated to fill out a consent form, and the INTERVAL study received approval from The National Research Ethics Service (11/EE/0538). The relevant data can be accessed openly at https://gwas.mrcieu.ac.uk. Statistics on digestive system tumors were collected from various GWAS databases, the International Classification of Diseases Tenth Revision codes(ICD10), definitions, diagnostic criteria or methods involving six digestive disorders are listed in **Additional File 2**. The genetic variation data for HCC and PCa were publicly accessible at https://www.ebi.ac.uk/gwas. HCC comprised of 475,638 samples (379 cases and 475,259 controls) and 24,194,938 single-nucleotide polymorphisms (SNPs). PCa comprised of 476,245 samples (1,196 cases and 475,049 controls) and 24,195,229 SNPs. The genetic variation data for BTC, CRC, and GC can be accessed openly at https://www.finngen.fi/en/access_results, BTC consists of 218,792 samples (109 cases and 218,683 controls) with 16,380,466 SNPs. CRC consists of 218,792 samples (3,022 cases and 215,770 controls) with the same number of SNPs. GC consists of 218,792 samples (633 cases and 218,159 controls) with the same number of SNPs. The genetic variation data for EC can be obtained openly from https://gwas.mrcieu.ac.uk, it includes 372,756 samples (740 cases and 372,016 controls) with 8,970,465 SNPs. The data presented in this study were derived exclusively from European population samples. These samples were obtained from independent GWAS databases, ensuring minimal overlap and bias. It is important to note that every participant granted informed written consent, and all studies underwent review and approval by the institutional ethical review boards of the relevant institutions. Therefore, no additional ethical approvals or licenses were necessary for this MR study.

### 2.2 Study design and selection of IVs

**Figure 1:**
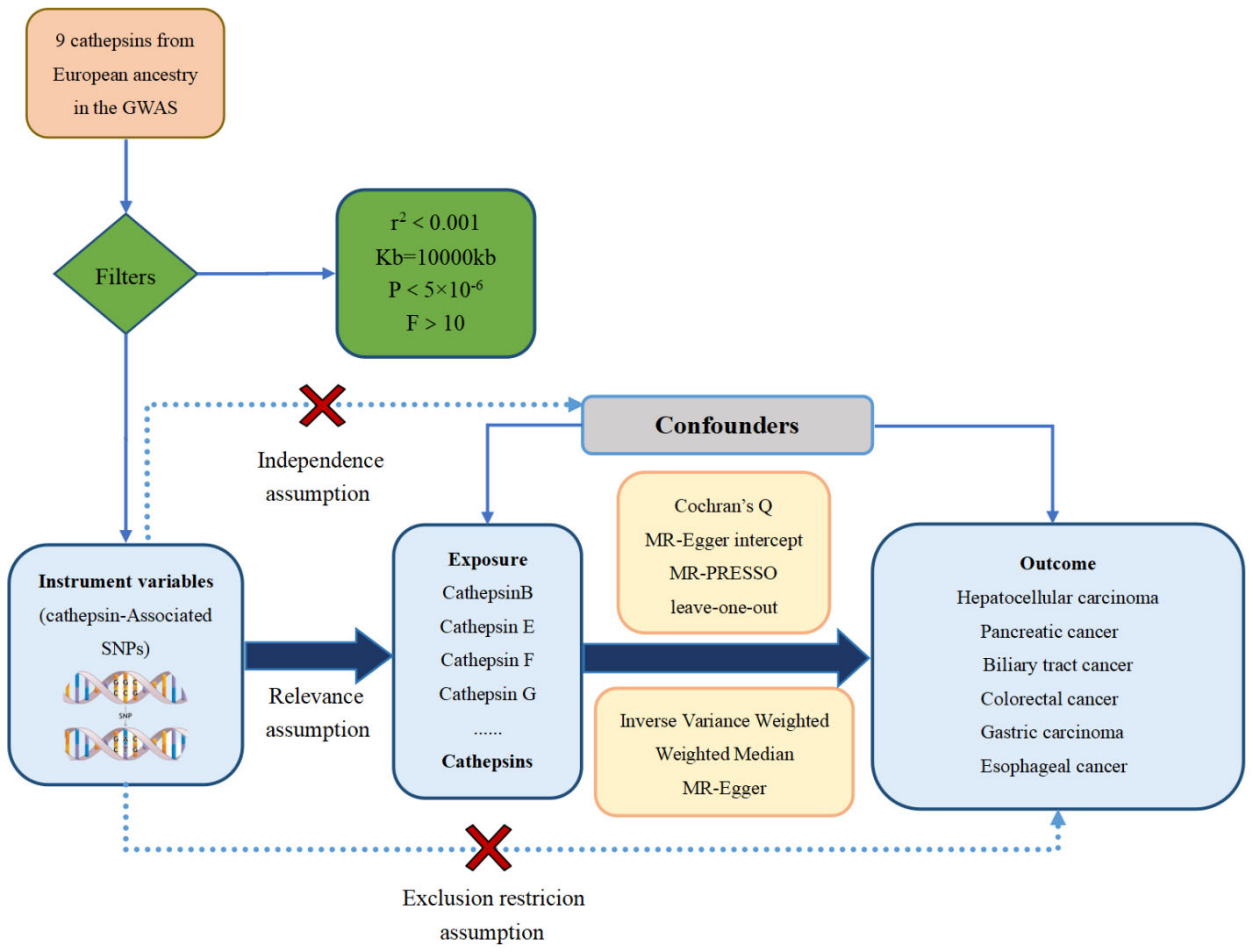
The three major hypotheses of two-sample Mendelian randomization and the research flow chart of the causal relationship between cathepsins and digestive system tumors.

The study methods were compliant with the STROBE-MR checklist (25), further details can be found in **Additional File 1**. These IVs needed to satisfy three core assumptions (26): the hypothesis of correlation, the hypothesis of exclusivity, and the assumption of independence, as illustrated in **Figure 1**. The first assumption establishes a robust link between SNPs and the variable of exposure. To identify SNPs associated with exposure factors and establish the validity and accuracy of the causal relationship between cathepsins and digestive system tumors, the following steps were followed to select the most suitable SNPs. Firstly, due to the restricted pool of SNPs accessible for MR analysis, a significance threshold of P value was less than 5×10^-6^ was established for the detection of SNPs that exhibit strong associations with the investigated exposures. Moreover, to eliminate any presence of linkage disequilibrium (27), an r^2^ threshold of 0.001 and a clump window size of 10,000 kb were implemented (27). Secondly, the selected SNPs were ensured to have no association with any confounding factors that could influence the relationship between exposure and outcome. Lastly, the SNPs were confirmed to only impact the outcome through exposure factors.

In addition, the selected IVs were assessed for the weak IV bias by calculating the F-statistic (28). The F-statistic for each SNP was calculated using the formula F=R2 (N−K−1)/[K(1−R2)] , where R^2^, N, and K represent the estimated exposure variance explained by the IVs, sample size, and the number of IVs (29). If the F-statistic was less than 10, the SNP was considered a weak IV and excluded from the analysis to mitigate bias caused by weak IVs.

## 3 MR analysis

In this study, two samples were analyzed bidirectionally by MR. Initially, the researchers examined the causal relationship between nine cathepsins and six digestive system tumors, with cathepsins considered as the exposure and digestive system tumors as the outcome. Subsequently, a reverse analysis was conducted using the same settings and data sets as the forward MR analysis. All analyses were conducted using R version 4.2.2, with the software packages ‘Two-SampleMR’ and ‘MR-PRESSO’ (30). Three analysis methods were employed in this study: Inverse variance weighting (IVW), MR-Egger, and weighted median (WM). The IVW method, considered the primary method for assessing causality (31), yielded a nominally significantly correlated result when the P value was less than 0.05. The IVW method is a classical method for MR analysis, where the weighted average is calculated by taking the reciprocal of the variance of each IV as the weight, ensuring the effectiveness of all IVs. To ensure the robustness of the MR results, both MR-Egger and WM methods were employed as complementary approaches. MR-Egger utilizes a weighted linear regression analysis, providing robust estimates that are independent of the validity of instrumental variables. Nevertheless, it is crucial to acknowledge that these estimates may have lower statistical precision and can be influenced by outlier genetic variation (32). On the other hand, The problem of estimation accuracy variability is tackled by the WM approach. In a manner reminiscent of the IVW approach, the WM method assigns inverse weights that are contingent upon the variance of individual genetic variants, demonstrating reliability even when causal effects are violated (33). The Cochran’s Q test was used to estimate the heterogeneity of SNPs. A p-value greater than 0.05 indicated the absence of heterogeneity. In the case of significant heterogeneity in SNPs, a random effects model was applied. Conversely, a fixed effects model was utilized instead. Additionally, to ensure the reliability of the results, a leave-one-out analysis was carried out. This analysis aimed to remove SNPs that could have potentially extreme effects (30). To identify horizontal pleiotropy, the MR-egger intercept was utilized. A p-value greater than 0.05 indicated no horizontal pleiotropy. If the p-value was less than 0.05 in situations, the outlier test was employed to eliminate horizontal pleiotropy by implementing the MR-PRESSO global test (34). Causality was evaluated using the odds ratio (OR) and 95% confidence interval (CI). If the OR value was less than 1, the exposure will be regarded as a protective factor for the outcome. Conversely, if the OR value was greater than 1, the exposure will be classified as a risk factor for the outcome. To visualize the MR analysis, forest plots, scatter plots, and leave-one-out plots were generated using the data analysis function of the Rstudio platform.

## 4 Results

### 4.1 IV selection

The genetic variants utilized in our study to analyze 9 cathepsins were sourced from the INTERVAL study, while statistical data for 6 digestive system tumors were gathered from various GWAS databases. By employing genome-wide significance threshold screening (P<5×10^-6^), multiple SNPs were identified as IVs for each cathepsin and disease in the bidirectional MR analysis. Each selected IV had an F-statistic exceeding 10, indicating the absence of weak IV bias. Detailed information on the SNPs can be found in **Supplementary Table** (1, 3).

### 4.2 MR main analysis results

**Figure 2:**
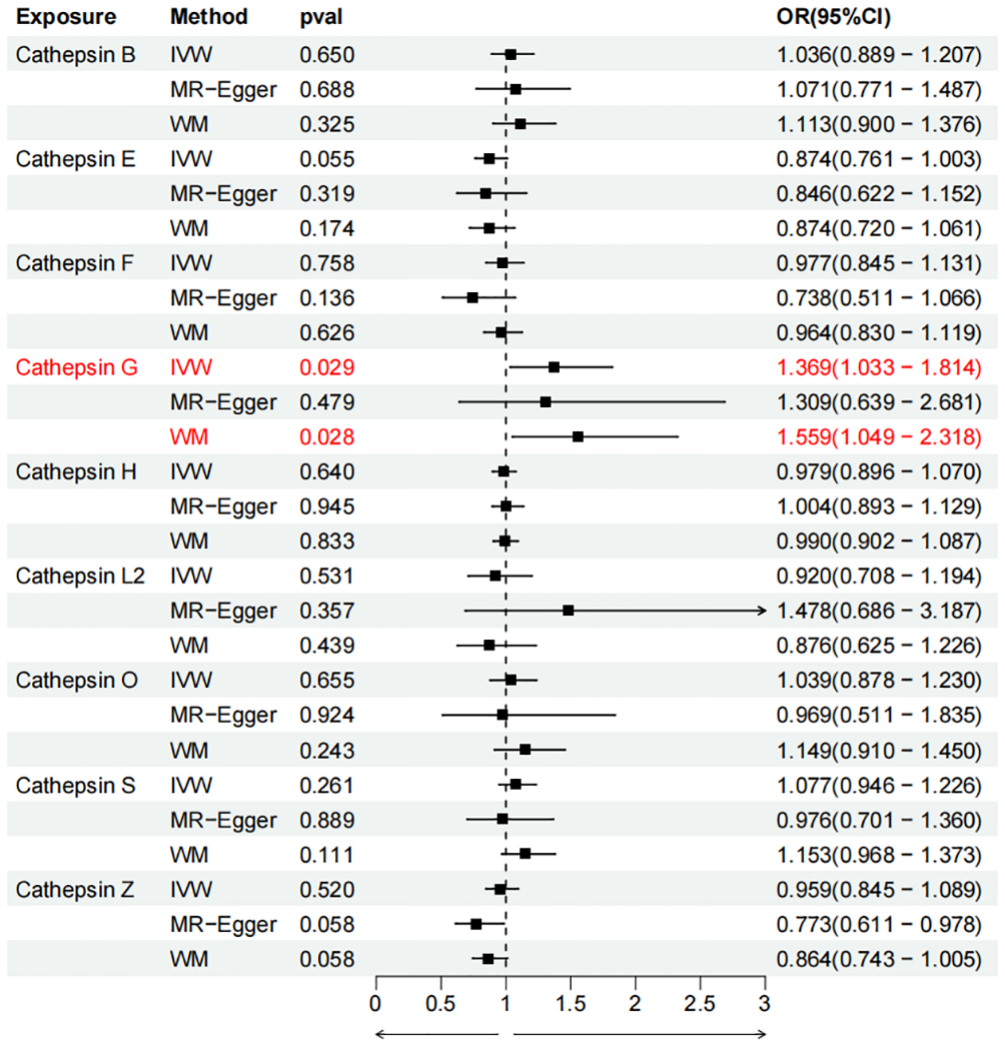
Forest plots of cathepsins on hepatocellular carcinoma. The forward causality were marked red in the figure. IVW, inverse variance weighting; WM, weighted median; OR, odds ratio; CI, confidence interval.

**Figure 3:**
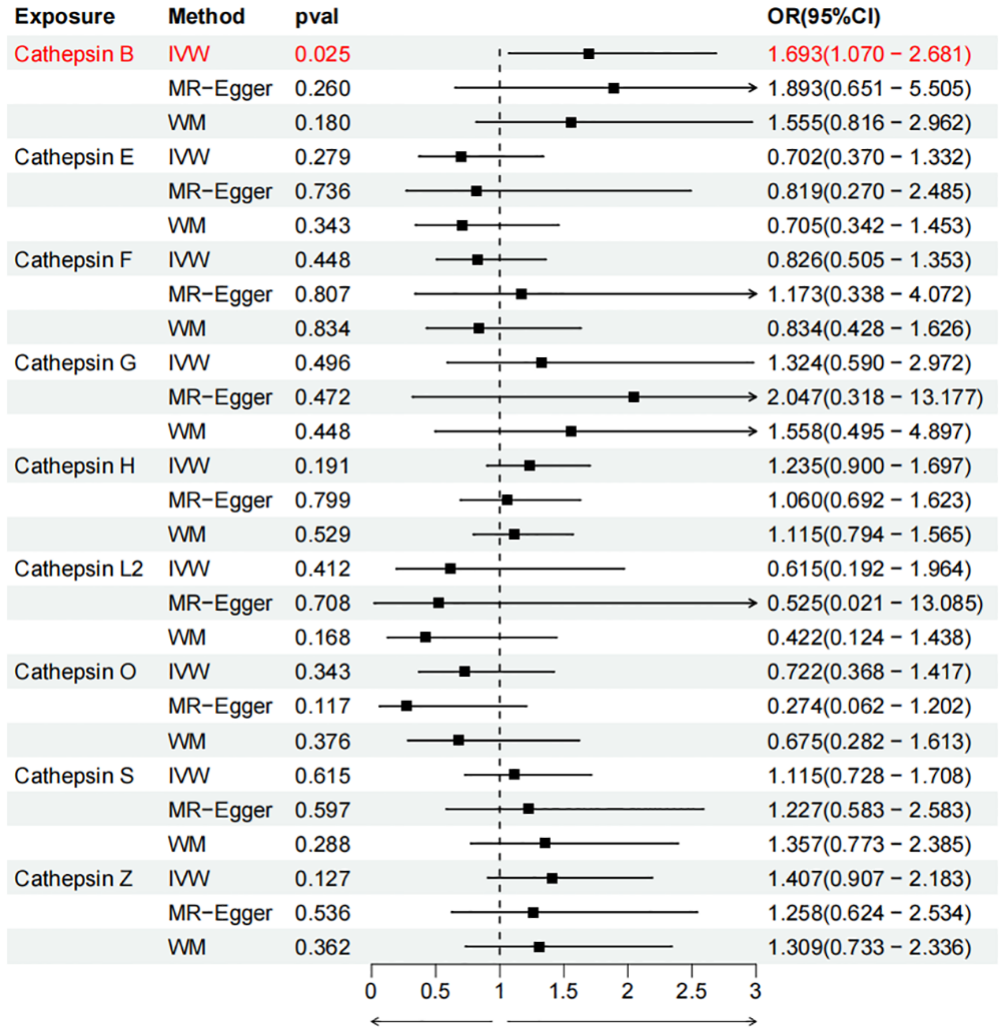
Forest plots of cathepsins on biliary tract cancer. The forward causality were marked red in the figure. IVW, inverse variance weighting; WM, weighted median; OR, odds ratio; CI, confidence interval.

**Figure 4:**
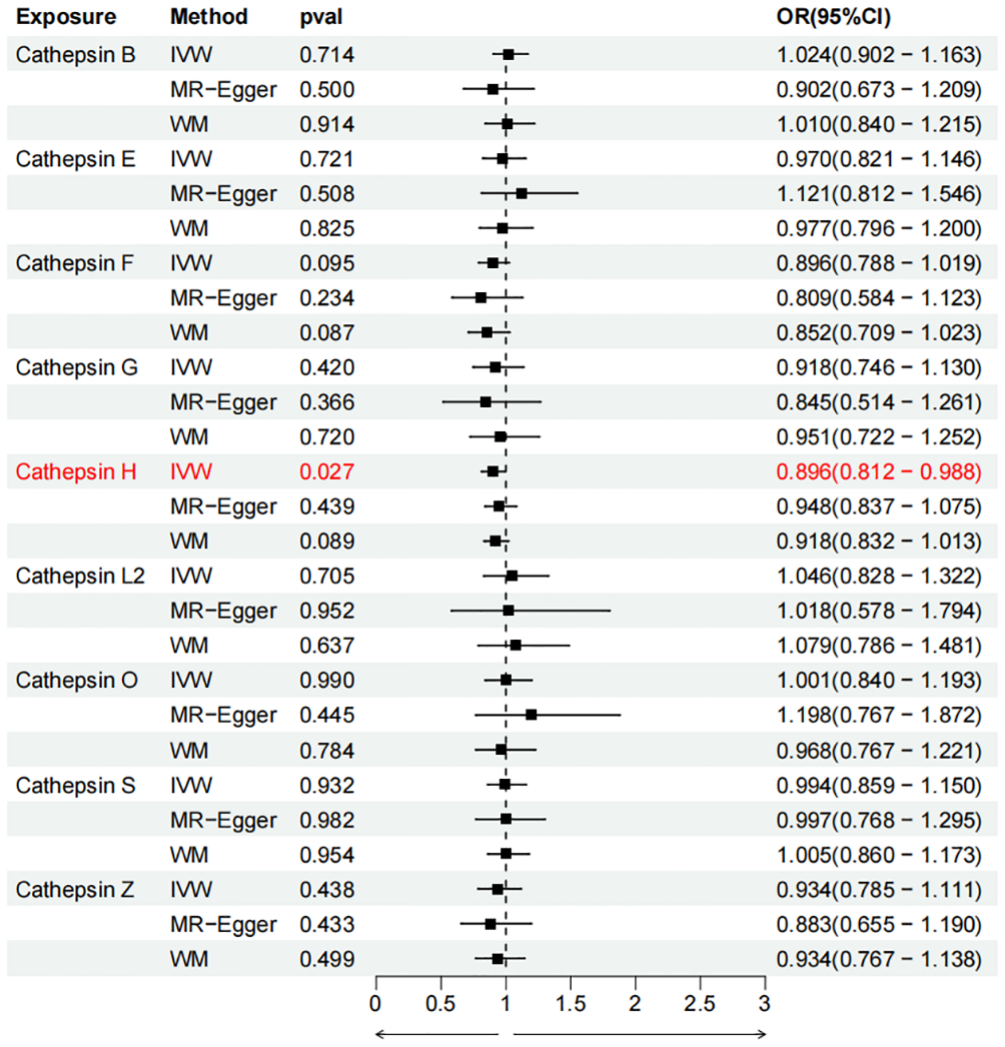
Forest plots of cathepsins on pancreatic cancer. The forward causality were marked red in the figure. IVW, inverse variance weighting; WM, weighted median; OR, odds ratio; CI, confidence interval.

**Figure 5:**
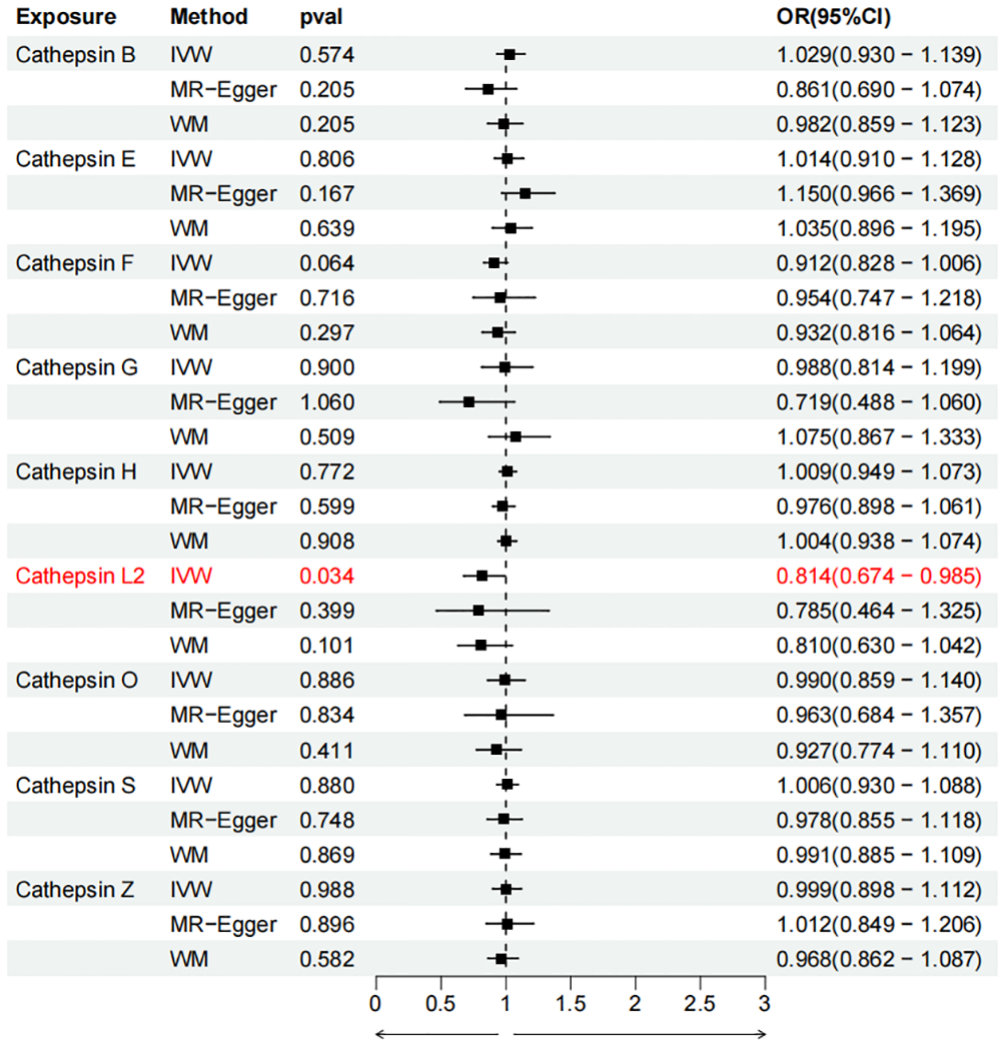
Forest plots of cathepsins on colorectal cancer. The forward causality were marked red in the figure. IVW, inverse variance weighting; WM, weighted median; OR, odds ratio; CI, confidence interval.

To evaluate the impact of various cathepsins on the risk of different types of digestive system tumors, a Two-SampleMR analysis was conducted. This analysis encompassed a total of nine cathepsins include cathepsin B, cathepsin E, cathepsin F, cathepsin G, cathepsin H, cathepsin L2, cathepsin O, cathepsin S, and cathepsin Z, and six digestive system tumors (HCC, PCa, BTC, CRC, GC, and EC). This study utilized the IVW method as the primary analysis technique. IVW is a method used for meta-analyzing the effects of multiple genetic variants, which can generate reliable causal estimates without the presence of directional pleiotropy. The findings from the univariate MR analysis indicated a causal relationship between four cathepsins and four digestive system tumors (**Table 1**). Specifically, The risk of HCC increased with high levels of cathepsin G (IVW: p = 0.029, odds ratio (OR) = 1.369, 95% CI = 1.033-1.814), the forest plot is shown in **Figure 2**. Similarly, BTC was associated with elevated cathepsin B levels (IVW: p = 0.025, OR = 1.693, 95% CI = 1.070-2.681), the forest plot is shown in **Figure 3**. Conversely, a reduction in PCa risk was associated with increased cathepsin H levels (IVW: p = 0.027, OR = 0.896, 95% CI = 0.812-0.988), the forest plot is shown in **Figure 4**. Lastly, high levels of cathepsin L2 were found to lower the risk of CRC (IVW: p = 0.034, OR = 0.814, 95% CI = 0.674-0.985), the forest plot is shown in **Figure 5**. The IVW results indicated that cathepsin G was associated with a 36.9% increase in the risk of HCC (OR = 1.369), cathepsin B was linked to a 69.3% increase in the risk of BTC (OR = 1.693), while cathepsin H was found to reduce the risk of PCa by 10.4% (OR = 0.896), and cathepsin L2 was associated with an 18.6% reduction in the risk of CRC (OR = 0.814). The presence of a causal association between digestive system tumors and other forms of cathepsins was not found by employing the IVW method (**Supplementary Table 1**).

**Table 1:** Mendelian randomization analysis with forward causality.

Cathepsin	outcome	Inverse variance weighted		MR-Egger		Weighted median	
		OR (95%CI)	p_value	OR (95%CI)	p_value	OR (95%CI)	p_value
Cathepsin B	BTC	1.693(1.070-2.681)	0.025	1.893(0.651-5.505)	0.260	1.555(0.816-2.962)	0.180
Cathepsin G	HCC	1.369(1.033-1.814)	0.029	1.309(0.639-2.681)	0.479	1.559(1.049-2.318)	0.028
Cathepsin H	PCa	0.896(0.812-0.988)	0.027	0.948(0.837-1.075)	0.439	0.918(0.832-1.013)	0.089
Cathepsin L2	CRC	0.814(0.674-0.985)	0.034	0.785(0.464-1.325)	0.399	0.810(0.630-1.042)	0.101

### 4.3 MR sensitivity analyses results

**Figure 6:**
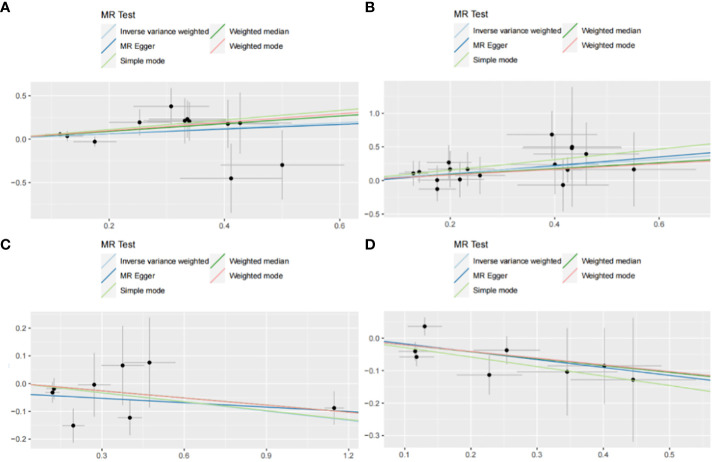
Scatter plot with forward causality in Mendelian randomization. **(A)** Cathepsin G on hepatocellular carcinoma. **(B)** Cathepsin B on biliary tract cancer. **(C)** Cathepsin H on pancreatic cancer. **(D)** Cathepsin L2 on colorectal cancer.

**Figure 7:**
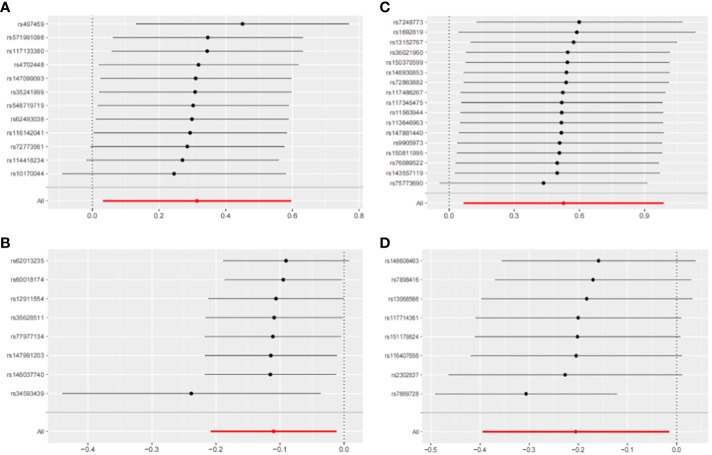
Leave-one-out sensitivity analysis with forward causality in Mendelian randomization. **(A)** Cathepsin G on hepatocellular carcinoma. **(B)** Cathepsin B on biliary tract cancer. **(C)** Cathepsin H on pancreatic cancer. **(D)** Cathepsin L2 on colorectal cancer.

The IVW method may overlook potential pleiotropic effects, therefore we conducted sensitivity analyses such as MR-Egger and weighted median to evaluate the reliability and consistency of the findings. Additionally, The Cochran’s Q test was used to estimate the heterogeneity of SNPs. A p-value greater than 0.05 indicated the absence of heterogeneity. To identify horizontal pleiotropy, the MR-egger intercept was utilized. A p-value greater than 0.05 indicated no horizontal pleiotropy. In **Supplementary Table 2**, both Cochran’s Q and MR-Egger intercepts did not indicate any evidence of heterogeneity or pleiotropy in these causal relationships. The scatter plot is shown in **Figure 6**. The results of the leave-one-out analysis demonstrate the robustness of these findings, as illustrated in **Figure 7**.

### 4.4 Reverse MR analysis

To address potential reverse causation, we conducted a reverse Two-SampleMR analysis using 6 digestive system tumors as exposures and 9 cathepsins as outcomes. The results of the MR analysis did not show any evidence of reverse causality (**Supplementary Table 3**).

## 5 Discussion

The occurrence and progression of tumors in the digestive system entail a remarkably intricate procedure, wherein proteolytic occurrences assume indispensable functions. A mounting quantity of investigations has been concentrating on scrutinizing the function of cathepsins in tumors of the digestive system (35). In this study, we employed genetic instruments to systematically examine the causal relationship between nine cathepsins and six digestive system tumors. Through two-sample bidirectional MR analysis, we observed potential associations between four cathepsins and four digestive system tumors. Specifically, higher levels of cathepsin G were found to increase the risk of HCC, while elevated levels of cathepsin B were linked to an increased risk of BTC. Conversely, elevated levels of cathepsin H were found to potentially decrease the risk of PCa, and Increased levels of cathepsin L2 were correlated with a potential decrease in the risk of CRC. These findings suggest that different cathepsins may have varying effects on different types of digestive system tumors.

The conclusion of the study further clarified the previously observed correlation between cathepsin B and BTC (18, 19), indicating a potential causal relationship. Cathepsin B, a cysteine protease, acts mainly as an endopeptidase in the normal cellular endolysosomal compartment. However, during tumor development, the regulation of cathepsin B may suffer disturbances at different stages, leading to its excessive expression and subsequent release into the extracellular space. Cathepsin B has an important function in controlling tumor growth, migration, invasion, angiogenesis, and metastasis, particularly in digestive system tumors (36). Moreover, previous studies have demonstrated the substantial impact of cathepsin B on the growth of HCC (15). However, the present MR analysis does not establish a causal relationship between the two variables, suggesting the involvement of intricate mechanisms that require further investigation.

Activated neutrophils secrete Cathepsin G, which is a serine protease and is closely associated with tumor diseases (37). The interaction of cathepsin G, found on neutrophils, with the receptor for advanced glycation end products (RAGE), found on the surface of tumors, plays a critical role in the cancer-fighting abilities of neutrophils (38). Furthermore, cathepsin G can enhance the adhesion and migration of cancer cells (39), as well as facilitate tumor formation by regulating tumor-matrix interactions (40). Increasing numbers of studies have shown that neutrophil extracellular traps (NET) are associated with tumor progression. In the process, cathepsin G, in conjunction with matrix metalloproteinase 9 (MMP-9) and neutrophil elastase (NE), actively participates (41). Previous experimental studies have confirmed that the interplay of tumor cells and neutrophils promotes the metastasis of liver cancer through the cathepsin G component related to NET (42), indicating a correlation between cathepsin G and HCC. This study strengthens the evidence of a causal link between cathepsin G and HCC, implying the potential of cathepsin G as a target for therapeutic intervention in HCC. These findings provide valuable insights for the treatment of HCC.

The present study unveiled a previously unmentioned finding that cathepsin H reduces the risk of PCa. Cathepsin H belongs to the papain superfamily of lysosomal cysteine proteases and is the sole known aminopeptidase in this family (43). It is synthesized as an inactive precursor and becomes activated through protein hydrolysis to remove its prepeptide (44). Cathepsin H has been reported to be associated with cancer and other major diseases. However, there is an insufficiency of existing investigations concerning the correlation between cathepsin H and PCa. Additional research is imperative to acquire a comprehensive understanding of the intricate linkage between the two entities. The results of the present MR analysis serve as a valuable reference for future studies on cathepsin H and PCa. Previous studies have indicated that several cathepsins are linked to the occurrence and progression of PCa (45). Specifically, cathepsin E has been found to have high expression and activity in pancreatic ductal adenocarcinoma (PDAC) (46), while cathepsin B has been shown to play a significant role in the autonomous growth of PDAC, potentially promoting tumor cell proliferation through the regulation of cathepsin L (16). Nevertheless, the MR analysis conducted in this study failed to establish a conclusive causal link between cathepsin E, cathepsin B, and the development of PCa.

According to MR analysis, the presence of cathepsin L2 has been found to reduce the risk of CRC. Cathepsin L2, also known as cathepsin V, is a human lysosomal cysteine peptidase that plays specific roles in pathological mechanisms and is crucial in the breakdown of the extracellular matrix (47). Cathepsin L2 is involved in the release of antigenic peptides, the maturation of major histocompatibility complex (MHC) class II molecules, the turnover of elastinogen fibers, as well as the cleavage of intracellular and extracellular substrates (48). It has been discovered that cathepsin L2, which is widely expressed in human tumors, plays an essential part in promoting the proliferation of bladder cancer by increasing NF-κB activity (9). Furthermore, it has been found that cathepsin L2 can also drive the progression of lung cancer by influencing the immunosuppressive environment and the cleavage of adhesion molecules (47). Furthermore, the activity of cathepsin L2 has been demonstrated to govern the advancement of the cell cycle and maintain the stability of histones within the nucleus of malignant cells found in breast cancer (49). It is also closely associated with the prognosis of HCC and the tumor microenvironment (50). Interestingly, this study reveals that cathepsin L2 reduces the risk of CRC, which has not been reported in previous studies. This suggests a potentially more complex relationship between cathepsin L2 and CRC, and further research is required to comprehend the underlying mechanism of action.

Previous research have highlighted the importance of cathepsin B in the development, invasion, and metastasis of CRC (21). Furthermore, the expression of cathepsin S in CRC has been reported to be substantial (51). However, the current MR analysis did not establish a causal relationship between cathepsin B, S, and CRC. Apart from BTC, HCC, PCa, and CRC as mentioned earlier, there are also relevant reports suggesting that cathepsin B serves as a sensitive indicator for gastrointestinal malignant tumors (52). This indicates the potential importance of cathepsin B in digestive system tumors, which warrants further investigation.

Tumor screening is gaining more attention in the prevention of tumor diseases. The screening of serum biomarkers is also becoming more convenient and efficient. The objective of this investigation is to examine the causal relationship between various cathepsins and different digestive system tumors through MR analysis. The study employs genetic variations as a means to minimize the impact of confounding factors and reverse causal connections. Using genetics as a foundation for analysis helps to establish a stronger correlation between variables and reduce the likelihood of unrelated factors affecting the results. This approach allows for a more reliable and accurate understanding of the data, as it circumvents the potential biases that may be introduced by external factors. By focusing on genetic variations, the study aims to provide a clearer picture of the relationships under investigation and offers a valuable contribution to the field. The findings of this study can help in the search for effective tumor markers and provide potential value for further research on digestive system tumors. Nevertheless, it is critical to recognize the constraints of our investigation. Firstly, the databases used in the study only included individuals of European ancestry. To obtain stronger evidence, it is necessary to expand the databases to include other ethnic groups such as those from Asia and Africa. Secondly, the threshold of P value was less than 5×10^-8^ is generally considered to indicate genome-wide significance when screening for instrumental variables. In this study, we aimed to increase the number of SNPs to mitigate bias from a limited number of IVs, therefore, we consulted relevant literature on Mendelian randomization of cathepsin and established a significance threshold of P value less than 5×10^-6^. Nevertheless, caution is advised when interpreting the results. Third, the MR analysis method is a theoretical causal analysis method that requires further validation through animal experiments to establish the causal relationship. This will help in understanding the intricate mechanism linking cathepsins and digestive system tumors.

## 6 Conclusion

In conclusion, the results demonstrate a potential causal relationship between specific cathepsins and digestive system tumors. These findings may offer potential targets and new biomarkers for the diagnosis and treatment of digestive system tumors. However, further interventional trials are needed to clarify the underlying mechanisms.

## Data availability statement

The original contributions presented in the study are included in the article/**Supplementary Material**. Further inquiries can be directed to the corresponding author.

## Author contributions

XH: Conceptualization, Data curation, Formal Analysis, Investigation, Methodology, Project administration, Resources, Software, Supervision, Validation, Visualization, Writing – original draft, Writing – review & editing. HD: Methodology, Writing – review & editing. BZ: Data curation, Writing – review & editing. KW: Data curation, Writing – review & editing. YQ: Visualization, Writing – review & editing. TL: Project administration, Writing – review & editing. TJL: Supervision, Writing – review & editing.
