# Supplementary material for: The causal relationship between cathepsins and digestive system tumors: a Mendelian randomization study
Source: Front Oncol. 2024 Mar 25;14:1365138. doi: 10.3389/fonc.2024.1365138 (PMC10999587; doi:10.3389/fonc.2024.1365138)
Supplement: Supplementary file 1 [file DataSheet_1.pdf]

STROBE-MR checklist of recommended items to address in reports of Mendelian randomization studies[1 2]

Item No.	Section		Checklist item	Relevant text from manuscript
1	**TITLE and ABSTRACT**		Indicate Mendelian randomization (MR) as the study's design in the title and/or the abstract if that is a main purpose of the study	The causal relationship between cathepsins and digestive system tumors：a Mendelian randomization study.
	**INTRODUCTION**			
2	**Background**		Explain the scientific background and rationale for the reported study. What is the exposure? Is a potential causal relationship between exposure and outcome plausible? Justify why MR is a helpful method to address the study question	Multiple studies have confirmed the significant role of cathepsins in the development and progression of digestive system tumors. However, further investigation is needed to determine the causal relationships.
3	**Objectives**		State specific objectives clearly, including pre-specified causal hypotheses (if any). State that MR is a method that, under specific assumptions, intends to estimate causal effects	Our research goal is to confirm the causal relationship between cathepsins and digestive system tumors and provide valuable insights for the diagnosis and treatment of digestive system tumors.
	**METHODS**			
4	**Study design and data sources**		Present key elements of the study design early in the article. Consider including a table listing sources of data for all phases of the study. For each data source contributing to the analysis, describe the following:	
		a)	Setting: Describe the study design and the underlying population, if possible. Describe the setting, locations, and relevant dates, including periods of recruitment, exposure, follow-up, and data collection, when available.	The MR analysis of nine cathepsin levels in this investigation obtained the genetic tools from the INTERVAL study, which comprised 3301 Europeans. The relevant data can be accessed openly at https://gwas.mrcieu.ac.uk. Statistics on digestive system tumors were collected from various GWAS databases. The genetic variation data for HCC and PCa were publicly accessible at https://www.ebi.ac.uk/gwas. HCC comprised of 475,638 samples (379 cases and 475,259 controls) and 24,194,938 SNPs. PCa comprised of 476,245 samples (1,196 cases and 475,049 controls) and 24,195,229 SNPs. The genetic variation data for BTC, CRC, and GC can be accessed at https://www.finngen.fi/en/access_results, BTC consists of 218,792 samples (109 cases and 218,683 controls) with 16,380,466 SNPs. CRC consists of 218,792 samples (3,022 cases and 215,770 controls) with the same number of SNPs. GC consists of 218,792 samples (633 cases and 218,159 controls) with the same number of SNPs. The genetic variation data for EC can be obtained openly from

			https://gwas.mrcieu.ac.uk, it includes 372,756 samples (740 cases and 372,016 controls) with 8,970,465 SNPs.
		b) Participants: Give the eligibility criteria, and the sources and methods of selection of participants. Report the sample size, and whether any power or sample size calculations were carried out prior to the main analysis	Between mid-2012 and mid-2014, blood donors aged 18 years and older were recruited at 25 centres of England's National Health Service Blood and Transplant (NHSBT). All participants gave informed consent before joining the study and the National Research Ethics Service approved this study (11/EE/0538). Participants completed an online questionnaire including questions about demographic characteristics (for example, age, sex, ethnicity), anthropometry (height, weight), lifestyle (for example, alcohol and tobacco consumption) and diet. nation criteria exclude people with a history of major diseases (such as myocardial infarction, stroke, cancer, HIV, and hepatitis B or C) and those who have had recent illness or infection.
		c) Describe measurement, quality control and selection of genetic variants	To identify SNPs associated with exposure factors and establish the validity and accuracy of the causal relationship between cathepsins and digestive system tumors, the following steps were followed to select the most suitable SNPs. Firstly, due to the restricted pool of SNPs accessible for MR analysis, a significance threshold of P value was less than 5×10^{-6} was established for the detection of SNPs that exhibit strong associations with the investigated exposures. Moreover, to eliminate any presence of linkage disequilibrium, an r^2 threshold of 0.001 and a clump window size of 10,000 kb were implemented. In addition, the selected IVs were assessed for the weak IV bias by calculating the F-statistic.
		d) For each exposure, outcome, and other relevant variables, describe methods of assessment and diagnostic criteria for diseases	Detailed information on disease assessment methods and diagnostic criteria is provided in Additional file 2.
		e) Provide details of ethics committee approval and participant informed consent, if relevant	Informed consent was obtained from all participants, and the INTERVAL study received approval from The National Research Ethics<br>Service (11/EE/0538).
5	**Assumptions**	Explicitly state the three core IV assumptions for the main analysis (relevance, independence and exclusion restriction) as well assumptions for any additional or sensitivity analysis	In the MR analysis, SNPs were considered as IVs. These IVs needed to satisfy three core assumptions: the hypothesis of correlation, the hypothesis of exclusivity, and the assumption of Independence. The first assumption establishes a robust link between SNPs and the variable of exposure. Secondly, the selected SNPs were ensured to have no association with any confounding factors that could

				influence the relationship between exposure and outcome. Lastly, the SNPs were confirmed to only impact the outcome through exposure factors.
6	**Statistical methods: main analysis**		Describe statistical methods and statistics used	
		a)	Describe how quantitative variables were handled in the analyses (i.e., scale, units, model)	This research does not involve any transformations of quantitative variables.
		b)	Describe how genetic variants were handled in the analyses and, if applicable, how their weights were selected	Three analysis methods were employed in this study: Inverse variance weighting (IVW), MR-Egger, and weighted median (WM). The IVW method, considered the primary method for assessing causality, yielded a nominally significantly correlated result when the P value was less than 0.05. To ensure the robustness of the MR results, both MR-Egger and WM methods were employed as complementary approaches. The Cochran's Q test was used to estimate the heterogeneity of SNPs. Additionally, to ensure the reliability of the results, a leave-one-out analysis was carried out. To identify horizontal pleiotropy, the MR-egger intercept was utilized. Causality was evaluated using the odds ratio (OR) and 95% confidence interval (CI).
		c)	Describe the MR estimator (e.g. two-stage least squares, Wald ratio) and related statistics. Detail the included covariates and, in case of two-sample MR, whether the same covariate set was used for adjustment in the two samples	Genetic associations with all exposures were taken from a large meta-analysis of GWAS, we obtained SNP-specific Wald estimates and then used inverse variance weighting (IVW) with multiplicative random effects, MR-Egger, and weighted median (WM). The IVW method is a classical method for MR analysis, where the weighted average is calculated by taking the reciprocal of the variance of each IV as the weight, ensuring the effectiveness of all IVs. MR-Egger utilizes a weighted linear regression analysis, providing robust estimates that are independent of the validity of instrumental variables. Nevertheless, it is crucial to acknowledge that these estimates may have lower statistical precision and can be influenced by outlier genetic variation. On the other hand, The problem of estimation accuracy variability is tackled by the WM approach. In a manner reminiscent of the IVW approach, the WM method assigns inverse weights that are contingent upon the variance of individual genetic variants, demonstrating reliability even when causal effects are violated.
		d)	Explain how missing data were addressed	In this MR analysis, the issue of missing data was not

				involved.
		e)	If applicable, indicate how multiple testing was addressed	In this MR analysis, multiple exposures or multiple outcomes were not involved, so multiple testing was not performed.
7	**Assessment of assumptions**		Describe any methods or prior knowledge used to assess the assumptions or justify their validity	To assess the risk of weak instrument bias, the selected IVs were assessed for the weak IV bias by calculating the F-statistic.The F-statistic for each SNP was calculated using the formula $F = R^2 (N - K - 1) / [K (1 - R^2)]$. To investigate the degree of bias in the initial causal estimates due to pleiotropic effects, we used some sensitivity analyses, for example: MR-Egger, WM approach. MR-Egger and WM approach were implemented using the R package TwoSampleMR."
8	**Sensitivity analyses and additional analyses**		Describe any sensitivity analyses or additional analyses performed (e.g. comparison of effect estimates from different approaches, independent replication, bias analytic techniques, validation of instruments, simulations)	To ensure the robustness of the MR results, both MR-Egger and WM methods were employed as complementary approaches. MR-Egger utilizes a weighted linear regression analysis, providing robust estimates that are independent of the validity of instrumental variables. Nevertheless, it is crucial to acknowledge that these estimates may have lower statistical precision and can be influenced by outlier genetic variation. On the other hand, The problem of estimation accuracy variability is tackled by the WM approach. In a manner reminiscent of the IVW approach, the WM method assigns inverse weights that are contingent upon the variance of individual genetic variants, demonstrating reliability even when causal effects are violated. The Cochran's Q test was used to estimate the heterogeneity of SNPs. Additionally, to ensure the reliability of the results, a leave-one-out analysis was carried out. This analysis aimed to remove SNPs that could have potentially extreme effects. To identify horizontal pleiotropy, the MR-egger intercept was utilized.
9	**Software and pre-registration**			
		a)	Name statistical software and package(s), including version and settings used	All analyses were conducted using R version 4.2.2, with the software packages 'Two-SampleMR' and 'MR-PRESSO'. To visualize the MR analysis, forest plots, scatter plots, and leave-one-out plots were generated using the data analysis function of the Rstudio platform.
		b)	State whether the study protocol and details were pre-registered (as well as when	This study was not pre-registered with the study protocol

		and where)	and details.
	**RESULTS**		
10	**Descriptive data**		
	a)	Report the numbers of individuals at each stage of included studies and reasons for exclusion. Consider use of a flow diagram	The MR analysis of nine cathepsin levels in this investigation obtained the genetic tools from the INTERVAL study, which comprised 3301 Europeans. Every contributor was obligated to fill out a consent form, and the INTERVAL study received approval from The National Research Ethics Service (11/EE/0538). Statistics on digestive system tumors were collected from various GWAS databases. HCC comprised of 475,638 samples. PCa comprised of 476,245 samples. BTC consists of 218,792 samples. CRC consists of 218,792 samples. GC consists of 218,792 samples. EC includes 372,756 samples.
	b)	Report summary statistics for phenotypic exposure(s), outcome(s), and other relevant variables (e.g. means, SDs, proportions)	Summary data on exposure and outcomes are shown in Table 1 and Supplementary Tables (1, 2, 3).
	c)	If the data sources include meta-analyses of previous studies, provide the assessments of heterogeneity across these studies	The Cochran's Q test was used to estimate the heterogeneity of SNPs, detailed data are provided in Supplementary Tables (2, 3).
	d)	For two-sample MR:<br>i. Provide justification of the similarity of the genetic variant-exposure associations between the exposure and outcome samples<br>ii. Provide information on the number of individuals who overlap between the exposure and outcome studies	The data presented in this study were derived exclusively from European population samples. These samples were obtained from independent GWAS databases, ensuring minimal overlap and bias, detailed data on the number of individuals in the exposure and outcome samples are provided in Table 1 and Supplementary Tables (1, 3).
11	**Main results**		
	a)	Report the associations between genetic variant and exposure, and between genetic variant and outcome, preferably on an interpretable scale	The risk of HCC increased with high levels of cathepsin G (IVW: p = 0.029, odds ratio (OR)= 1.369, 95% confidence interval (CI) = 1.033-1.814). Similarly, BTC was associated with elevated cathepsin B levels (IVW: p = 0.025, OR = 1.693, 95% CI = 1.070-2.681). Conversely, a reduction in PCa risk was associated with increased cathepsin H levels (IVW: p = 0.027, OR = 0.896, 95% CI = 0.812-0.988). Lastly, high levels of cathepsin L2 were found to lower the risk of CRC (IVW: p = 0.034, OR = 0.814, 95% CI = 0.674-0.985).
	b)	Report MR estimates of the relationship between exposure and outcome, and the measures of uncertainty from the MR analysis, on an interpretable scale, such as	Mengelian randomization estimation reports are detailed in

			odds ratio or relative risk per SD difference	Table 1, Supplementary Tables (1,3).
		c)	If relevant, consider translating estimates of relative risk into absolute risk for a meaningful time period	The calculation of absolute risk is detailed in Table 1, Supplementary Tables (1,3).
		d)	Consider plots to visualize results (e.g. forest plot, scatterplot of associations between genetic variants and outcome versus between genetic variants and exposure)	The results are visualized in Figure (2-7).
12	**Assessment of assumptions**			
		a)	Report the assessment of the validity of the assumptions	Firstly, we selected the SNPs of cathepsin as instrumental variables, which have a strong association with digestive system tumors, allowing us to perform Mendelian randomization inferences, and the large F statistics indicate that these analyzes will not be affected by weak instrument bias. Secondly, the selected SNPs were ensured to have no association with any confounding factors that could influence the relationship between exposure and outcome. Lastly, the SNPs were confirmed to only impact the outcome through exposure factors.
		b)	Report any additional statistics (e.g., assessments of heterogeneity across genetic variants, such as I^2, Q statistic or E-value)	The Cochran's Q test did not detect any heterogeneity of the SNPs, These causal relationships did not show any directional pleiotropy according to the MR-Egger intercept test, as detailed in Supplementary Tables (2,3).
13	**Sensitivity analyses and additional analyses**			
		a)	Report any sensitivity analyses to assess the robustness of the main results to violations of the assumptions	IVW proves the causal relationship between cathepsin G, B, H, L2 and digestive system tumors. The WM method supports the above causal relationship that elevated levels of cathepsin G increase the risk of liver cancer, but the WM method and the MR-Egger method did not further confirm the relevance of the above other IVW analyses, as detailed in Table 1, Supplementary Tables (1,3).
		b)	Report results from other sensitivity analyses or additional analyses	Leave-one-out sensitivity analysis demonstrated the robustness of the MR results, as detailed in FIGURE 7.
		c)	Report any assessment of direction of causal relationship (e.g., bidirectional MR)	We used bidirectional MR analysis, The forward analysis proved the causal relationship between cathepsin G, B, H, L2 and digestive system tumors, as detailed in Table 1 and

				Supplementary Table 1. However, no reverse causal relationship between cathepsins and digestive system tumors was found. See Supplementary Table 3 for details.
		d)	When relevant, report and compare with estimates from non-MR analyses	This study does not involve non-MR studies.
		e)	Consider additional plots to visualize results (e.g., leave-one-out analyses)	To visualize the MR analysis, forest plots, scatter plots, and leave-one-out plots were generated using the data analysis function of the Rstudio platform, as detailed in FIGURE (2-7).
	**DISCUSSION**			
14	**Key results**		Summarize key results with reference to study objectives	Our findings confirm the causal relationship between cathepsin G, B, H, L2 and digestive system tumors, Specifically, higher levels of cathepsin G were found to increase the risk of HCC, while elevated levels of cathepsin B were linked to an increased risk of BTC. Conversely, elevated levels of cathepsin H were found to potentially decrease the risk of PCa, and Increased levels of cathepsin L2 were correlated with a potential decrease in the risk of CRC.
15	**Limitations**		Discuss limitations of the study, taking into account the validity of the IV assumptions, other sources of potential bias, and imprecision. Discuss both direction and magnitude of any potential bias and any efforts to address them	Firstly, the databases used in the study only included individuals of European ancestry. To obtain stronger evidence, it is necessary to expand the databases to include other ethnic groups such as those from Asia and Africa. Secondly, the threshold of P value was less than 5×10^{-8} is generally considered to indicate genome-wide significance when screening for IVs. However, in this study, the threshold of P value was set less than 5×10^{-6} in order to obtain a sufficient number of SNPs. It is important to interpret the study results with caution, as this difference in threshold may have some impact on the findings. Third, the MR analysis method is a theoretical causal analysis method that requires further validation through animal experiments to establish the causal relationship. This will help in understanding the intricate mechanism linking cathepsins and digestive system tumors.
16	**Interpretation**			
		a)	Meaning: Give a cautious overall interpretation of results in the context of their limitations and in comparison with other studies	The results demonstrate a potential causal relationship between specific cathepsins and digestive system tumors.
		b)	Mechanism: Discuss underlying biological mechanisms that could drive a potential causal relationship between the investigated exposure and the outcome, and whether	Cathepsins are lysosomal proteolytic enzymes that are responsible for maintaining cellular homeostasis. They

		the gene-environment equivalence assumption is reasonable. Use causal language carefully, clarifying that IV estimates may provide causal effects only under certain assumptions	primarily function as endopeptidases within the lysosomal vesicles of normal cells. Cathepsins are involved in various physiological processes such as protein turnover, differentiation, and apoptosis. They also play important roles in signaling cellular stress, breaking down the extracellular matrix, causing lysosome-mediated cell death, and have been associated with the progression of a diversity of diseases, including malignancies.
	c)	Clinical relevance: Discuss whether the results have clinical or public policy relevance, and to what extent they inform effect sizes of possible interventions	These findings may offer potential targets and new biomarkers for the diagnosis and treatment of digestive system tumors.
17	**Generalizability**	Discuss the generalizability of the study results (a) to other populations, (b) across other exposure periods/timings, and (c) across other levels of exposure	This study provides a comprehensive analysis of the causal relationship between cathepsins and digestive system tumors. However, it did not investigate the effects of varying exposure periods or levels. Furthermore, the study was limited to a European population, raising questions about its generalizability to other populations.
	**OTHER INFORMATION**		
18	**Funding**	Describe sources of funding and the role of funders in the present study and, if applicable, sources of funding for the databases and original study or studies on which the present study is based	There is no funding for this research.
19	**Data and data sharing**	Provide the data used to perform all analyses or report where and how the data can be accessed, and reference these sources in the article. Provide the statistical code needed to reproduce the results in the article, or report whether the code is publicly accessible and if so, where	Details are provided in the data availability statement and supplementary materials.
20	**Conflicts of Interest**	All authors should declare all potential conflicts of interest	The authors declare that the research was conducted in the absence of any commercial or financial relationships that could be construed as a potential conflict of interest.

This checklist is copyrighted by the Equator Network under the Creative Commons Attribution 3.0 Unported (CC BY 3.0) license.

1. Skrivankova VW, Richmond RC, Woolf BAR, Yarmolinsky J, Davies NM, Swanson SA, et al. Strengthening the Reporting of Observational Studies in Epidemiology using Mendelian Randomization (STROBE-MR) Statement. JAMA. 2021;under review.
2. Skrivankova VW, Richmond RC, Woolf BAR, Davies NM, Swanson SA, VanderWeele TJ, et al. Strengthening the Reporting of Observational Studies in Epidemiology using Mendelian Randomisation (STROBE-MR): Explanation and Elaboration. BMJ. 2021;375:n2233.
