# Supplementary material for: The causal relationship between cathepsins and digestive system tumors: a Mendelian randomization study
Source: Front Oncol. 2024 Mar 25;14:1365138. doi: 10.3389/fonc.2024.1365138 (PMC10999587; doi:10.3389/fonc.2024.1365138)
Supplement: Supplementary file 2 [file DataSheet_2.pdf]

1 hepatocellular carcinoma

1.1 Code of International Classification of Diseases: C22.9

1.2 Disease definition: Hepatocellular carcinoma (HCC) is a malignant tumor originating from liver cells.

1.3 Diagnostic criteria or methods: HCC can be diagnosed if any one of the following three conditions is met. (1) There are two typical imaging manifestations of liver cancer (US, contrast-enhanced CT, MRI or selective hepatic arteriography), and the lesion is >2cm. (2) A typical imaging finding of HCC: lesions >2cm and AFP >400ng/ml. (3) Positive liver biopsy.

2 pancreatic cancer

2.1 Code of International Classification of Diseases: C25.9

2.2 Disease definition: Pancreatic cancer (PCa) refers to a malignant tumor that mainly originates from pancreatic duct epithelium and acinar cells.

2.3 Diagnostic criteria or methods: B-ultrasound examination is the preferred diagnostic method for pancreatic cancer. The diagnosis of tumors larger than 2cm can be confirmed, and pancreatic duct dilation, intrahepatic or/and extrahepatic dilation of bile ducts, gallbladder enlargement and intrahepatic metastasis can also be found. CT scan can show the surrounding conditions of pancreatic tumors, the location and size of pancreatic tumors, etc, and can detect tumors about 1cm in diameter.

3 biliary tract cancer

3.1 Code of International Classification of Diseases: C22.1--C24.0

3.2 Disease definition: Biliary tract cancer(BTC) is a malignant tumor that occurs in the gallbladder or intrahepatic and extrahepatic bile ducts.

3.3 Diagnostic criteria or methods: Positive imaging findings and significantly elevated tumor markers can make a preliminary diagnosis. Surgery and postoperative pathology can make a clear diagnosis of gallbladder cancer. Endoscopic ampullary biopsy can help make a clear diagnosis of cholangiocarcinoma.

4 colorectal cancer

4.1 Code of International Classification of Diseases: C18.9

4.2 Disease definition: Colorectal cancer (CRC) refers to malignant tumors that occur in the colon and rectum, mainly adenocarcinoma.

4.3 Diagnostic criteria or methods: Colonoscopy and pathological examination of mucosal biopsy can confirm the diagnosis.

5 gastric carcinoma

5.1 Code of International Classification of Diseases: C16.9

5.2 Disease definition: Gastric carcinoma (GC) refers to malignant tumors originating from gastric mucosal epithelial cells, and the vast majority are adenocarcinomas.

5.3 Diagnostic criteria or methods: Gastroscopy combined with mucosal biopsy is currently the most reliable diagnostic method.

6 esophageal cancer

6.1 Code of International Classification of Diseases: C15.9

6.2 Disease definition: Esophageal cancer (EC) is a malignant tumor originating from the esophageal mucosal epithelium, mainly squamous cell carcinoma and adenocarcinoma.

6.3 Diagnostic criteria or methods: The preferred method for esophageal cancer is endoscopy, in which endoscopic biopsy can confirm the diagnosis. In addition, CT examination can clarify the anatomical relationship between the esophagus and adjacent mediastinal organs, the extent of tumor invasion, and metastatic lesions.
